# Precision dentistry—what it is, where it fails (yet), and how to get there

**DOI:** 10.1007/s00784-022-04420-1

**Published:** 2022-03-14

**Authors:** Falk Schwendicke, Joachim Krois

**Affiliations:** Department of Oral Diagnostics, Digital Health and Health Services Research Charité – Universitätsmedizin Berlin, Aßmannshauser Str. 4-6, 14197 Berlin, Germany

**Keywords:** Data, Predictive modeling, Personalized medicine, Risk assessment, Systems medicine

## Abstract

**Objectives:**

Dentistry is stuck between the one-size-fits-all approach towards diagnostics and therapy employed for a century and the era of stratified medicine. The present review presents the concept of precision dentistry, i.e., the next step beyond stratification into risk groups, and lays out where we stand, but also what challenges we have ahead for precision dentistry to come true.

**Material and methods:**

Narrative literature review.

**Results:**

Current approaches for enabling more precise diagnostics and therapies focus on stratification of individuals using clinical or social risk factors or indicators. Most research in dentistry does not focus on predictions — the key for precision dentistry — but on associations. We critically discuss why both approaches (focus on a limited number of risk factors or indicators and on associations) are insufficient and elaborate on what we think may allow to overcome the status quo.

**Conclusions:**

Leveraging more diverse and broad data stemming from routine or unusual sources via advanced data analytics and testing the resulting prediction models rigorously may allow further steps towards more precise oral and dental care.

**Clinical significance:**

Precision dentistry refers to tailoring diagnostics and therapy to an individual; it builds on modelling, prediction making and rigorous testing. Most studies in the dental domain focus on showing associations, and do not attempt to make any predictions. Moreover, the datasets used are narrow and usually collected purposively following a clinical reasoning. Opening routine data silos and involving uncommon data sources to harvest broad data and leverage them using advanced analytics could facilitate precision dentistry.

## Precision and prediction

Based on a limited understanding of the complexity of dental diseases, oral and dental care has been managing conditions like dental caries or periodontitis in a similar way for all patients and disease stages for centuries [1]. For example, carious lesions have usually been managed via a restorative, i.e., invasive approach, building on the removal of all carious tissue and the restoration of the cavities, without further consideration for lesion stages, alternative management options, or the wider environment leading to the lesion development in the first place. Similarly, periodontal disease has been primarily managed by the removal of supra- and sub-gingival calculus (deep scaling and root planing) without further consideration of the lesion depth, a more staged management approach or the general determinants of the disease. The same can be said for diagnostic pathways; in many instances, all patients received a visual-tactile evaluation including pocket probing as well as radiographs to screen for both caries and periodontitis in identical intervals and modes, regardless of their previous disease experience and further aspects to be considered. In summary, dentistry followed a “one-size-fits-all” approach in both diagnostics and therapy.

Building on a deeper understanding of oral conditions, their pathogenesis and trajectories, and, most importantly, the acknowledgement of the wider social, behavioral, and systemic determinants of oral health as well as their link with general health [2], a far broader set of diagnostic and therapeutic strategies has been developed over the last decades. For caries, for example, non- and micro-invasive strategies are by now routinely available in many settings to manage different lesion stages; these interventions are also applied to maintain health and prevent disease initiation or progression [3]. For periodontal diseases, the control and modulation of the oral microbiome as well as the management of periodontal inflammation are increasingly in the focus [4].

The application of these wide ranged interventions, however, continues to follow a simplified set of decision-making triggers like the presumed activity of the carious lesion or periodontal site, its stage or depth, and the general extent of the condition in the oral cavity. The assignment of interventions on patient level similarly builds on a limited number of risk indicators or risk factors (Box) which dentists are supposed to employ to stratify patients, e.g., into low, medium, or high risk (Table 1). Dentistry has, so far, not succeeded in paying tribute to the deep and broad differences between individuals.
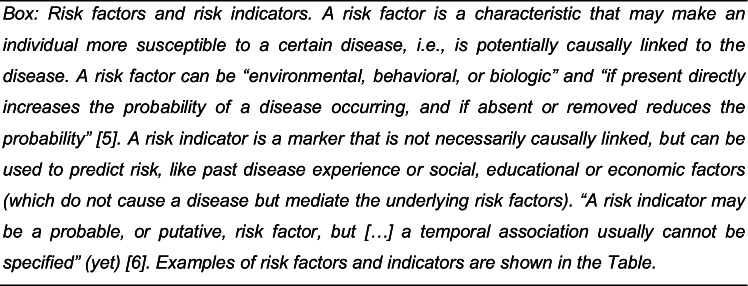


**Table 1:** Exemplary risk factors and risk indicators for the main oral conditions, caries and periodontitis

	Caries	Periodontitis
Risk factor		
Diet	Sugar	Sugar
Other behavior	Oral hygiene, fluoridated toothpaste	Oral hygiene, smoking
Biomarkers	Bacterial composition	Bacterial composition, genetic factors (SNPs)
Risk indicator		
Past disease experience	Caries experience	Periodontitis experience
Status	Low social, educational or economic status	Low social, educational or economic status
Medical status	Medication causing hyposalivation/xerostomia	Medication inducing immunosuppression

**Fig. 1:**
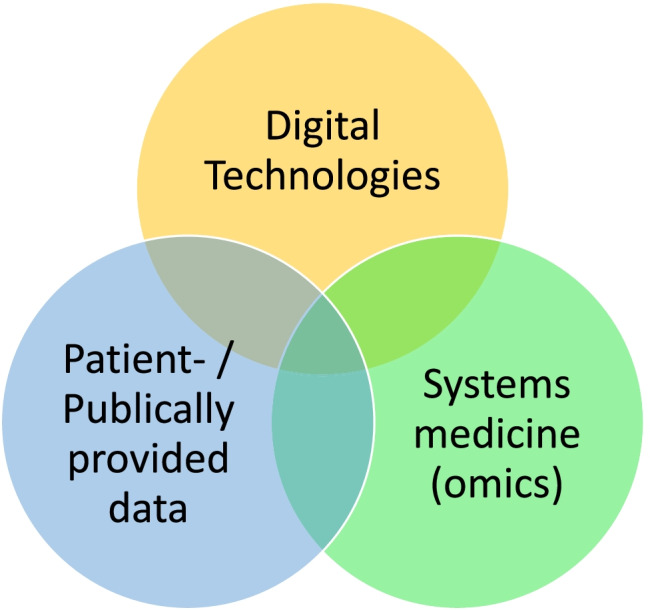
P4 medicine. Precise (more effective and efficient, safer) and personalized (targeted and individualized), but also preventive (early intervention, ideally before disease onset) and participatory (involving the patient, e.g., as data donor or recipient) care is facilitated by a range of factors: Digital technologies (including compute and storage hardware, but also software), data from systems medicine (mainly omics data) and data provided by patients or social networks, but also publicly available risk indicators

This is where precision medicine comes into play: Precision medicine refers to the tailoring of a therapy to an individual, i.e., his or her biological (genomic, microbiomic, proteomic), social (economic, educational), and behavioral (lifestyle) characteristics or traits, allowing to predict which therapy may be most efficacious, efficient, and safe, but also to prevent the onset and progression of early disease stages. Precision medicine in other fields like oncology increasingly expands beyond this to include the adaptation of the therapy itself to an individual. Notably, precision medicine should, and increasingly does, involve diagnostics, i.e., the adaptation of the wealth of available diagnostic strategies to each individual. Precision medicine is closely linked with another term, P4 medicine: a more precise and personalized, but also preventive and participatory approach towards healthcare (Fig. 1). All of these aspects build on the idea of predicting what will happen to a patient or a specific organ or site; precision medicine is closely linked with medicine becoming more predictive.

With an ever-increasing amount of diagnostic and therapy options being available also in dentistry, we should experience the advent of a more precise, personalized dentistry, too. However, the reality is different; oral and dental care continues to focus on stratifying individuals in risk groups, while the tools employed for this stratification often lack validity and accuracy, as discussed in detail below.

In addition, oral and dental research circles around associations, assuming these will allow stratification or, in the long-term, prediction-based precise care. We will, over the following sections of this article, explain why association studies may only limitedly advance the field of precision dentistry, and will display the status quo of predictive approaches in dentistry. We will then explain why we may need a more data-centric approach to facilitate precision dentistry, and eventually discuss the challenges we face and what action points for the oral and dental health and research community we identify.

## Association is not predictive value

So far, and as mentioned, oral and dental research has either focused on establishing associations between risk factors/indicators and health outcomes in cross-sectional studies (e.g., smoking status and tooth loss), or has presented associations between risk factors/indicators and caries increment (new or progressing caries lesions in an individual), periodontal disease progression (new or progressing periodontal disease sites in an individual), caries or periodontal lesion progression on site or tooth level (lesion activity), or tooth loss in longitudinally followed populations. While the former type of study is unable to infer to any predictive value (as temporal links cannot be established), the latter type may allow to test for predictions. However, it is relevant to bear in mind that finding patterns and associations in a longitudinally followed sample does not mean one can predict anything—neither for this nor for any another sample: The minimum to demonstrate a predictive value would be to develop a model on a subset (training dataset) of this sample and then test it on a separate, independent second subset (hold-out test dataset) of this sample. Only when showing that what was learnt on a subset of data allows to predict an outcome on another (unseen) subset, one should claim predictive value.

A second, more ambitious, and even less often seen approach in dental research is demonstrating some degree of generalizability (also called transportability). Here, one would develop (train) a prediction model on one population and test it on another, fully independent population (from another center, or another time period etc.) [7]. Moreover, to demonstrate usefulness, prediction studies should not only interpret associations in relative terms (e.g., smoking increases the risk of periodontal disease progression by factor two), but in absolute terms (e.g., from 100 predictions towards patients’ future periodontal conditions, the model was accurate in 75 and inaccurate in 25 cases). Only then one can critically appraise the clinical applicability of the trained models.

So far, the wealth of association studies is not matched by a similar number of prediction studies, and as laid out testing of these prediction models has only been limitedly performed rigorously. Nevertheless, a range of prediction models have been developed for dental applications, with these models possibly allowing to move towards a more precise dental care. In the next section, we will discuss how useful these are.

## Prediction models used in dentistry

As laid out, prediction modeling is at the heart of precision dentistry. A range of prediction models have been developed: predicting (1) caries increment (number of new lesions or progressing ones on patient level, termed caries risk), (2) periodontal disease onset or progression (incidence of periodontitis, or worsening periodontal lesions or extent or stage on patient level, termed periodontitis risk); (3) progression of a specific caries lesion (termed caries lesion activity); (4) tooth loss (mainly in periodontitis patients and mainly during supportive periodontal care). All models are supposed to assist the practitioner in making more precise treatment planning decisions.

### Caries risk assessment

Knowledge about future caries increment would, in daily care, allow to assign specific interventions (e.g., tailoring a supportive oral program and deducing individualized intervals for supportive re-evaluations, targeting possible risk behaviors or traits). A wide range of models have been developed, building on both risk factors causally associated with disease incidence and progression, like diet, oral hygiene, or fluoride intake; and risk indicators like past caries experience (a surrogate for behavioral patterns, genetic or microbiomic traits), or socio-demographic status (a proxy for oral health literacy and behavior, among other aspects).

A recent review summarized the available tools [8]. In the review, 22 studies were included, five of them being of low risk of bias. All five studies assessed the Cariogram caries risk assessment tool, some of them additionally assessed other risk assessment instruments. Especially the full version of the Cariogram showed acceptable discrimination of individuals with versus those without disease onset and progression. The simplified version of Cariogram using fewer risk indicators or factors (e.g., omitting the assessment of salivary secretion rates, saliva buffering capacity, lactobacilli or *Streptococcus mutans* counts) was assessed by six studies, and showed similar acceptable discrimination. Other tools showed either lower discrimination (like CAMBRA) and/or were validated by only few (3 or less) studies. Notably, all tools employed similar risk indicators or factors; they mainly differed in the number and weighting of these.

### Periodontitis risk assessment

Like caries, having knowledge about an individual’s future periodontitis onset or progression, could help to tailor active periodontal care as well as the intensity and interval of supportive care. A systematic review from 2015 summarized the available tools [9]. Nineteen studies were included; six of them showed low risk of bias. A total of five risk assessment tools were identified. The most often investigated tool was the Periodontal Risk Assessment and its modifications, assessed by twelve studies. Five publications dealt with the DenPlan Excel/Previsor Patient Assessment and its modifications; the remaining tools were assessed by only 1–3 studies. Again, the different instruments employed a similar set of possible risk indicators or factors, while the number and weighting of them differed. The review stated that the instruments were able to discriminate individuals with different probability of disease progression, while one needs to highlight that overall, the validation of the instruments was limited, marred by high risk of bias and inconsistency. The review did not allow for synthesis and robust conclusions.

### Caries lesion activity

The assessment of specific lesions and their activity, i.e., progression risk, is also clinically relevant: A carious lesion can be active, i.e., suffer from ongoing demineralization and progression, or inactive, i.e., not demineralizing further and not progressing. Only few systems have been developed to assess lesion activity, most of them combining visual assessment (of lesion color, location, plaque coverage) with tactile evaluation (e.g., surface texture). A minority uses specific chemical or physical properties like the pH on a lesion, its fluorescent properties, or the loss of minerals itself. A recent systematic review identified 25 studies on this matter, with only very few instruments being validated by more than two studies (namely the Nyvad criteria, ICDAS-LAA and ICDAS-CAA) [10]. Validation was attempted on limited and not necessarily representative samples, focusing on diagnostic accuracy (discrimination) against a reference test (which is hard to establish here) or inter-examiner reliability, in some cases only on specific surfaces or teeth. The more common activity assessment systems showed moderate sensitivities and specificities, and low to moderate reliability.

### Tooth loss

Predicting tooth loss is a major goal in a range of fields, e.g., restorative dentistry, periodontology, and prosthodontics. Knowing which teeth may be not retained in a patient over the next decade could be useful to guide early therapy decisions, allowing more precise and efficient care. Notably, there are very few established prediction systems for this purpose; the vast majority of studies (for example in periodontology) assessed associations, not predictive value, as described [11].

In a recent study, we aimed to predict tooth loss on patient level (predicting how many, not which specific teeth were lost; for clinicians, the latter is more relevant) during supportive periodontal care across four university centers in Germany [12, 13]. Tooth loss in 897 patients was assessed, and prediction models were built on data of 75% of patients from one center and used for predictions on the remaining 25% of this center and 100% of data from the other three centers. The prediction error was assessed as root-mean-squared-error (RMSE), i.e., the deviation of predicted from actually lost teeth per patient and year. Annualized tooth loss/patient differed significantly between centers, and while age, smoking status and number of teeth at the beginning of supportive care were significantly associated with tooth loss, the median prediction errors ranged from 0.14 to 0.31, while the annual tooth loss was lower than 0.10 in all centers. In other words, none of the developed models was useful—and none was able to generalize to other centers! This study impressively showed that despite a large and diverse dataset being employed, and despite the known risk factors and indicators showing significant associations with the outcome, prediction making was not possible.

As mentioned, for a clinician, it would be more relevant to predict the loss of specific teeth (as this would allow individualized decision making, e.g., during active periodontal care). In another study, we aimed to assess if such tooth level predictions are feasible and if models of different complexity were more accurate in their prediction [14]. Again, data from periodontitis patients who had been followed long-term (up to > 25 years) in two university centers in Germany were used. Overall, tooth loss was a rare event (880 of 11,651 assessed teeth were lost) and hence hard to predict: While expected risk indicators like age, the number of teeth lost at baseline and teeth’s probing pocket depths were employed by the models for prediction making, even more complex (machine learning) models could not yield useful accuracy: It was plainly too hard — using the available set of covariates —to predict the 8% of teeth lost over the follow-up period. Moreover, and in line with the previous study discussed above, generalizability from one to another center was not given.

**Fig. 2:**
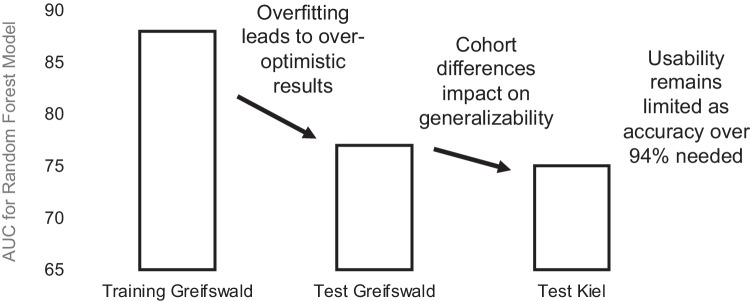
Area-under-the-curve (AUC) of models to predict tooth loss in periodontitis patients in two different centers (Greifswald and Kiel, Germany) from a recent prediction study [14]. A machine learning model (random forest) was used and trained on data from one and tested on data from the same and then the other center. The performance of a model on the training dataset is usually considerably higher than that on the test dataset, especially with more advanced models (which can learn the training data by heart, often referred to as “overfitting,” as it means the model perfectly fits to the training dataset but may be too specific to make useful predictions on any other dataset). When testing the model on the test dataset, performance drops. In addition, testing on data from another center (i.e., assessing generalizability) comes with further performance decreases, as cohorts differ. Last, and exemplified by this study, the prediction of scarce events is hard: In this particular study, even models with high performance were not more useful than guessing the majority class (in this case tooth retention), as the minority class (tooth loss) was highly infrequent. On such imbalanced datasets, even high accuracies may not be clinically useful

The findings of the latter studies are relevant and need highlighting, as they are common, but seldom communicated clearly (Fig. 2):

1. Finding statistically significant associations between risk factors or indicators and outcomes does by no means indicate that they allow to make accurate predictions.
2. Training on a subset of data and then testing the model on a hold-out test set is more reliable, but nevertheless oftentimes marred a range of problems, like the test set being small, drawn at random, and possibly suffering from data spoilage (also called data snooping bias, e.g., data from the same patient is spoiled in both the training and the test dataset).
3. Testing by cross-validation means training a range of models on several different partitions (subsets) of the data and then testing them on the remaining, different test partitions [15]. Cross-validation helps to overcome the described limits of a single hold-out test dataset, and also allows to reflect on the reliability and uncertainty of the models (as not one, but several models are essentially trained and tested).
4. Testing on a fully external population is even more rigorous and oftentimes leads to disappointment, as performance drops are particularly large here (given the limited generalizability of most models) [16].
5. And finally, building useful models when predicting scarce events over long time periods (e.g., tooth loss in maintained periodontitis patients) means we need to achieve accuracies higher than the so-called non-information rate (NIR). The NIR equals the chance that informed guessing is correct and reflects the majority class prevalence; for tooth loss in the described study, this rate was 92% — the vast majority of teeth which were not lost). Predictions will only be useful if their accuracy is higher than the NIR; in our case 92%. Researchers should consistently report their accuracy in association with the NIR. Also, other model performance metrics such as sensitivity and specificity or the confusion matrix should be additionally reported.

## Why we may fail

So why do prediction models in dentistry show only some usefulness (caries risk), unclear usefulness (periodontitis risk), or no usefulness at all (tooth loss) — after decades of research and more and more powerful software and hardware to make predictions? Why are we obviously quite some distance away from precision dentistry?

While it is not fully clear, one potential source of this problem lies with the risk factors and indicators we build these models on: They are either clinically determined and capture the phenotype of the unit of interest (a patient, a tooth, a surface, a pocket) or are recorded from patient history and questionnaires (on his or her diet, oral hygiene behavior etc.). In a nutshell, one could argue that oral and dental research has built models on those parameters that we as experts think are important. Moreover, we often employ parameters captured in a single time point (usually the baseline visit) and do not reflect changes in the status of risk indicators or factors over time.

In a recent study on claims data, we tried to overcome both aspects to some degree: In that study, we predicted mortality using predictor variables from a dataset of over 40,000 potential risk factors and indicators, many of them repeatedly collected from over 300,000 individuals (unpublished). Prediction was possible with high (and useful) accuracy. Notably, the most important risk indicators were not diseases or any specific treatments provided, but unexpected ones like the costs for transporting individuals or the fact that individuals consumed inpatient instead of outpatient care. As outlined below, using such large datasets, with longitudinally collected and broad data may allow to overcome the current constraints in prediction modeling. The technologies to harness these data (compute, algorithms, storage) are available; the main question, however, remains: Where to take the data from and what data could be used?

## Data dentistry

Data is considered a key resource for striving modern societies [17]. Many recent academic breakthroughs in astronomy [18], biology [19], and other disciplines are mostly and foremost driven by the analyses of huge data collections. Recently, we reflected on the transformational potential of rigorously applying data-centric principles in dentistry [20]. We promoted a shift in dentistry towards data-driven decision making and the dissemination of data-driven applications—a metamorphosis that we referred to as “data dentistry.” As a matter of fact, techniques and technologies needed to perform data-intensive science by now constitute the fourth paradigm for understanding nature, next to experimental and theoretical science and computer simulations [21]. As the costs of using data are low and decreasing over time, and data being able to be inexhaustibly used by as many agents as technologically feasible [22], it is increasingly seen as a key also for advancing healthcare, possibly facilitating a better, safer, more reliable, affordable, and accessible care.

Data-driven technologies are rapidly and irreversibly entering healthcare, for example, Artificial Intelligence (AI), advanced sensor technologies, including wearables, ingestibles, and implantables, and social media as well as and electronic health records (eHR), to name a few. These technologies are based on but also open up new sources of information for researchers and practitioners [23]. Many of these data sources will not solely rely on being collected in clinical settings, but routinely by patients, who may actively donate their data from social media, food consumption, healthcare apps, behavioral diaries, or toothbrushing patterns, among others, for medical purposes. These patients will become “partners” and actively participate in and contribute to their personalized medical journey. Together with claims data and clinical data stored in eHR that can be automatically categorized and mined using techniques of natural language processing, more precise and tailored treatments are in reach.

Another vastly unrecovered treasure trove of dense patient information are omics data. Notably, though, (earlier) studies gathering microbiomic, proteomic, or metabolomic data have been marred by limited replicability, and identified associations have — as laid out — not necessarily been confirmed as useful predictors, even if they were reproducible [24]. Moreover, the current model for relating omics data to health outcomes involves abstracting an identified relation from a modest number of patients evaluated in a research setting, and generalizing these abstractions (e.g., associations of genetic polymorphisms, microbiomic or metabolomic profiles with health outcomes). When these abstractions are considered to be reliable and useful, translation to the clinical setting is attempted, e.g., a prediction model and an associated clinical test are developed. This translational approach creates a large gap between the point of discovery (the research setting) and the point of care (the clinic), delaying innovations and leaving plenty of room for problems of accessibility, implementation, and maintenance decreasing the reach and, eventually, benefit of these discoveries to patients [23]. Gathering large, multimodal, and longitudinal omics data in routine settings, analyzing them using advanced data analytics technologies, and moving the “discovery step” of the translational pathway from the research lab to the clinical arena may increase the usefulness of omics-based applications, as any innovation is routed in clinical applicability straightaway [23].

**Fig. 3:**
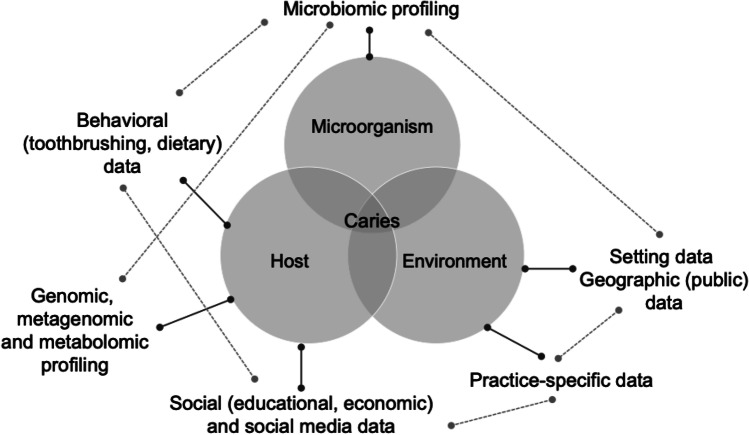
Along the traditional pillars of caries pathogenesis [30], a wide range of new data can be gathered, connected, and leveraged to yield a better understanding of the disease and to make individual predictions towards future caries risk of lesion activity. Various data sources collected from the host, the oral microbiome, or the environment (solid lines) are intertwined and related (dotted lines), resulting in a complex data lake which could be submitted to advanced data analytics for prediction modeling. Figure modified from [24]

Overall, we argue to employ data from a wide range of sources, not necessarily collected in a prospective and structured approach, but routinely and for other purposes, linking these data and submitting them to advanced data analytics for prediction making. For caries risk assessment, for example, combining the discussed data sources — all grounded in known contributors to the caries pathogenesis, but now being unraveled in unknown detail by technological advances — may allow the leap towards a truly precise dentistry (Fig. 3).

## Challenges ahead

A number of challenges remain before precision dentistry may come true:

1. Data availability: Dental data silos need to be broken up and made accessible for secure integration and use in research and clinical care. However, as data protection is a high good and legal frameworks and regulations impose well-defined guardrails, alternative approaches of sharing encrypted data or leveraging technologies such as federated learning [25], where data does not have to leave the actual physical site, need to be explored more thoroughly. More recently, Dayan et al. (2021) successfully applied federated learning techniques on data from 20 institutes across the globe to predict the future oxygen requirements of symptomatic patients with COVID-19 based on patients’ vital signs, laboratory data, and chest X-rays [26]. Their modelling approach improved generalizability and outperformed models that were trained at a single site using that site’s data. The availability of multi-center data and triangulation of data from different sources (imagery, text-based data, claims data, omics data, behavioral and environmental data, among others) may be the recipe missing, as discussed, of current approaches towards precision dentistry. In addition, calls for an active opt-out of data sharing and the implementation of broad consent concepts or options for efficient data donation have been raised, allowing to establish public datasets which could be used to benchmark prediction models.
2. Data linkage: Another main hurdle is lacking standards to enable data exchange and re-usage. Oral and dental data is usually not indexed properly (e.g., SNOMED: The Systematized Nomenclature of Medicine Clinical Terms, is not commonly used), and limited semantic interoperability means data cannot easily be exchanged, contrasted, or pooled. This may result in many medical data — if at all digitalized — to remain siloed despite being made theoretically accessible.
3. Bias, robustness, generalizability, and responsibility [27]. Many of the more advanced prediction models (which can leverage big and complex data) are black boxes, i.e., their inherent logic cannot be easily scrutinized for bias and reasoning. There are strong arguments for mandatory explainability of medical prediction models. Moreover, datasets used for predictive modeling should be appraised towards possible biases (e.g., sample selection bias), which impact on generalizability and fairness [28]. Bias may also originate from the users of any prediction model; confirmation and automation bias (or complacency) are phenomena where users follow any automated suggestion without sufficient attention, especially when challenged by multiple tasks at once [29]. Users should be educated about how to interpret the probabilistic output of prediction models and, generally, data literacy — as precision dentistry will be driven by data.
4. Resources and sustainability. Predictive modeling and precision dentistry will require huge amounts of data being stored, transmitted, and computed. The required technological resources have shown considerable growth rates over the past decades. Notably, the costs but also energy resources consumed for training and running these models are considerable and should be reflected, displayed, and appraised critically. Researchers and developers should routinely report on the consumed resources and both this consumption but also costs should be considered when evaluating technologies of precision dentistry.

## Conclusion

Precision dentistry refers to tailoring diagnostics and therapy to an individual, i.e., his or her biological (genomic, microbiomic, proteomic), social (economic, educational), and behavioral (lifestyle) characteristics or traits. Precision dentistry builds on modelling and prediction making. The data underlying any prediction are the key to successful precision dentistry. Most studies in the oral and dental domain focus on associations, and do not attempt to make any predictions. If predictions are made, they are often overly optimistic and do not reflect the true predictive power of the model, especially the prediction of scarce events (like tooth loss) remains a challenge. To overcome these limitations, the usage of broader and more diverse data — not necessarily reflecting what researchers feel may be relevant and not necessarily stemming from clinical studies, but routine — seems warranted. Challenges towards opening up and leveraging these routine data silos, using data in an efficient and sustainable way and critically appraising both the data but also any applications in the field of precision dentistry, remain ahead of us.

## Data Availability

N/a.
